# Constructing 3D microtubule networks using holographic optical trapping

**DOI:** 10.1038/srep18085

**Published:** 2015-12-10

**Authors:** J. Bergman, O. Osunbayo, M. Vershinin

**Affiliations:** 1 Department of Physics & Astronomy, Department of Biology, Center for Cell and Genome Science, University of Utah, 84112 Salt Lake City UT

## Abstract

Developing abilities to assemble nanoscale structures is a major scientific and engineering challenge. We report a technique which allows precise positioning and manipulation of individual rigid filaments, enabling construction of custom-designed 3D filament networks. This approach uses holographic optical trapping (HOT) for nano-positioning and microtubules (MTs) as network building blocks. MTs are desirable engineering components due to their high aspect ratio, rigidity, and their ability to serve as substrate for directed nano-transport, reflecting their roles in the eukaryotic cytoskeleton. The 3D architecture of MT cytoskeleton is a significant component of its function, however experimental tools to study the roles of this geometric complexity in a controlled environment have been lacking. We demonstrate the broad capabilities of our system by building a self-supporting 3D MT-based nanostructure and by conducting a MT-based transport experiment on a dynamically adjustable 3D MT intersection. Our methodology not only will advance studies of cytoskeletal networks (and associated processes such as MT-based transport) but will also likely find use in engineering nanostructures and devices.

Precisely constructed networks of rigid filaments are of broad interest. MTs are intriguing building blocks for nanoscale construction and mechanical engineering due to their small diameter, high rigidity and ability to sustain directed transport. For example, biomimetic engineering applications are starting to adopt MT-based motility to route and deliver nanoscale cargo^1^,^2^,^3^,^4^ but are severely limited by the lack of suitable nano-assembly techniques. Traditional *in vitro* approaches predominantly feature MTs fixed to a flat (typically coverslip) surface. Pillars and ridges on the coverslip surface have been used to produce suspended MTs that overhang freely or bridge several attachment points^5^,^6^. Such techniques are sufficient to sustain basic nano-transport but are neither flexible nor precise. Filaments cannot be positioned or re-positioned as needed, and 3D layouts are either impossible to achieve or highly restricted. Thus, existing approaches do not allow one to construct custom designed networks (whether the design is biologically inspired or technologically required).

Interest in constructing MT networks is further motivated by their importance in biological research. MT cytoskeletal networks in living cells are critical for tasks such as cell organization, and cargo transport. Despite this, the complexity of the 3D network and its role in cargo routing remains poorly characterized. It is often necessary to study cytoskeletal processes under controlled conditions^7^ but current *in vitro* techniques are unable to model complex 3D networks^8^,^9^. For example, cargo distribution within living cells cannot be fully understood without taking into account the cytoskeleton’s 3D nature e.g. 10,11. *In vivo* research is advancing rapidly to accommodate this need. Live cell 3D particle tracking techniques are steadily growing in number and sophistication e.g. 12,13,14. A live cell’s 3D MT network can now be visualized via super-resolution microscopy^15^,^16^, yet it is currently impossible to replicate the observed arrangement *in vitro*. Therefore, it is impossible to decouple the effects of intracellular regulation from the role of cytoskeletal topological complexity in a parallel *in vitro* study.

Our approach directly addresses these concerns. First, we demonstrate how to manipulate individual MTs in 3D. Refractive microspheres cross-linked to MTs serve as 3D positioning nodes which can be held and moved independently with HOTs. Key advantages of holography^17^ are scalability (hundreds of traps can be created and manipulated independently), 3D capability (traps can be defined anywhere within the accessible flow cell volume) and compatibility (often, HOT can be added to a system without modifying the pre-existing optical setup^18^). Prior related HOT-based techniques e.g. 19,20 served as an inspiration for our work but cannot be directly applied to MTs. Second, we show how to scale up our technique to assemble fully 3D MT networks, including efficient methodologies for assembling, storing, and integrating network building blocks. Finally, we demonstrate how our technique can be utilized to model and direct molecular motor transport by assembling 3D MT-MT crossings with dynamic control over filament angle and separation: features important for cargo routing^16^,^21^.

## Results

**Figure 1:**
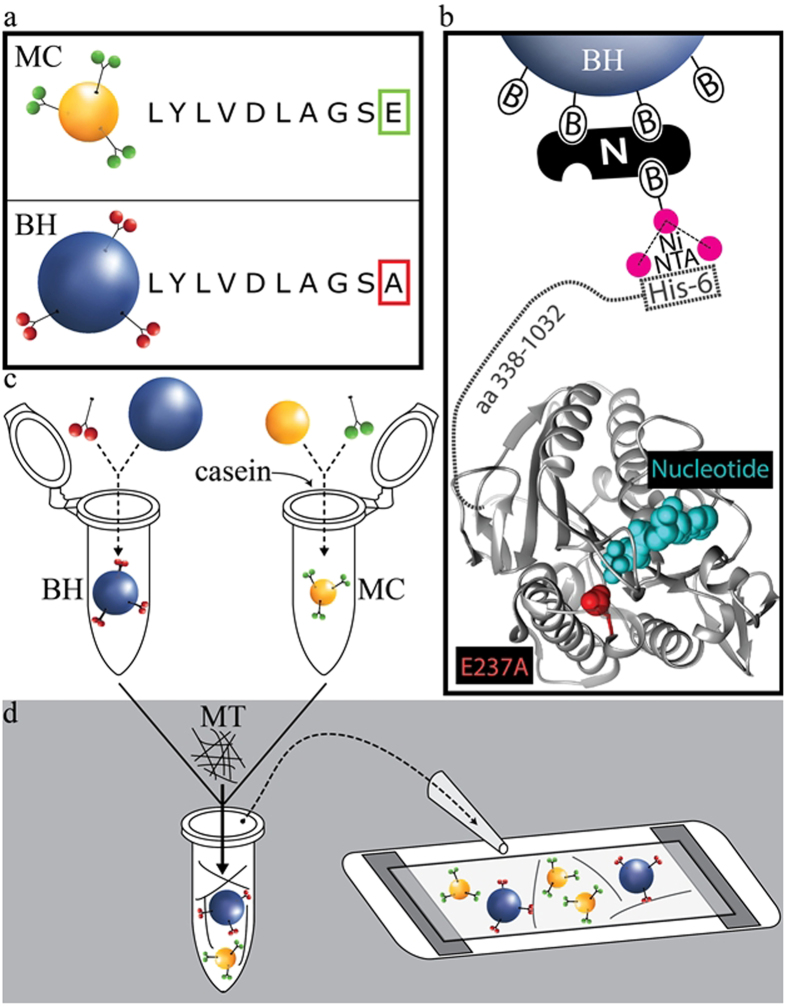
Basic BH-MT tethering strategy and preparation of assay constituents. (**a**) Motorized cargos are functionalized with WT hKIF5A while bead handles are functionalized with non-motile mutant hKIF5A (E237A). Construct sequences for kinesin-1 are otherwise identical, consisting of full length KHC and a HIS6 tag at the C-terminus. (**b**) BHs are specifically bound to E237A hKIF5A via a biotin-neutravidin-biotin sandwich (B-N-B in the figure) functionalized with a Nickel-activated tris-NTA molecule that binds with high affinity to the HIS6 tag on the E237A hKIF5A construct. Protein structure shows kinesin-1 motor domain. E237A mutation is highlighted in red and is in close proximity with a nucleotide (cyan). (**c**,**d**) BHs and MCs are prepared separately (see Methods), then combined together with MTs in assay buffer and finally are introduced into a flow chamber to allow filament network assembly.

**Figure 2:**
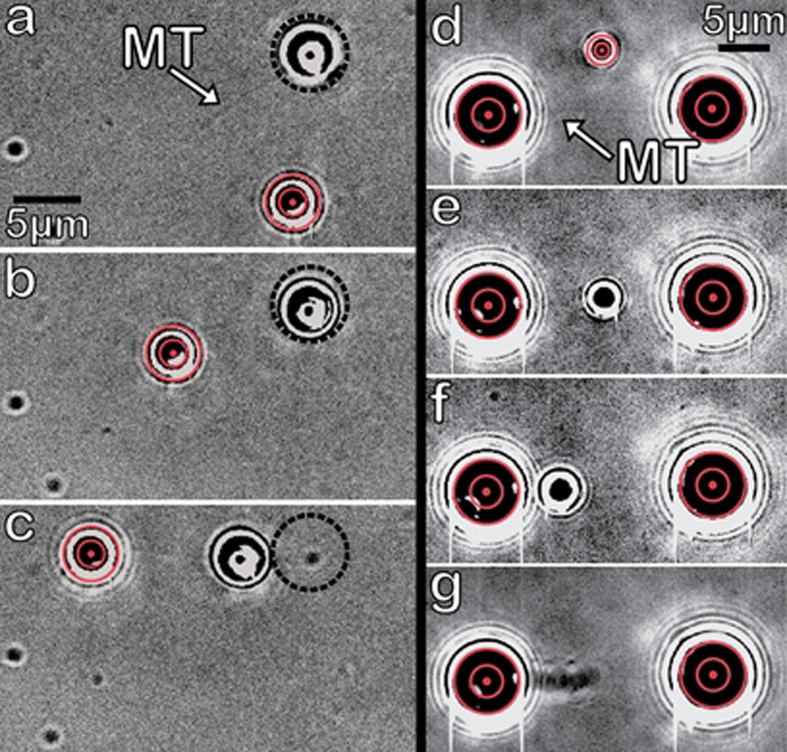
Dumbbell assembly and polarity characterization. (**a**) BH (highlighted by dotted black circle) starts out pre-attached to a MT and resting on the surface with second stand-alone BH close-by (red bulls-eye indicates it is being held in a HOT). (**b**) Trapped BH is maneuvered into close proximity to the “free” end of the MT. (**c**) MT attaches to trapped BH, and whole dumbbell assembly is moved by repositioning the HOT. Dotted black circle highlights original bead position. (**d**) A dumbbell is held taut by 2 HOTs, with a trapped MC nearby. (**e**) The MC is positioned to interact with the MT and is released upon binding. (**e**–**g**) The MC’s displacement indicates the MT’s polarity (here: plus end on the left). Movie frames corresponding to MC motion are averaged together to highlight that MC displacement is steady and continuous, rather than a result of detachment and reattachment.

**Figure 3:**
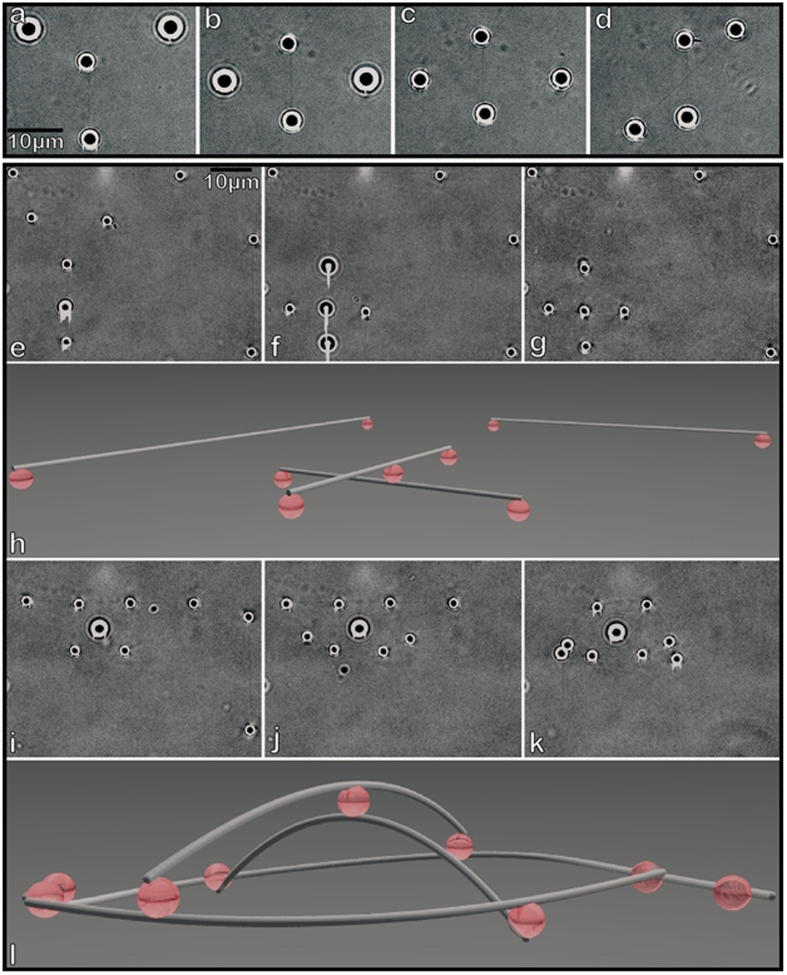
3D network assemblies. Demonstration of dynamic control of a MT intersection (**a**–**d**). 2 dumbbells are situated perpendicular to one another with the horizontal dumbbell raised relative to the vertical dumbbell by approximately 5 μm (**a**). The dumbbells are overlaid (**b**), then positioned at the same height (**c**), then reoriented to make a 45° angle (**d**), demonstrating the ability to adjust MT network geometry on demand. Assembly of a static 3D network (**e**–**l**). Four dumbbells are assembled, with one dumbbell featuring a BH attached in the middle (**e**). Two dumbbells are overlaid (**f**) and then bound together via the BH at the point of intersection (**g**). The 3D schematic of the resulting arrangement is shown in (**h**). The MT cross is reoriented and the outlying BHs are brought closer to the center until the central BH is displaced by ~3.5 μm out of the base plane (**i**). The apparent diameter of the central bead is larger due to the bead being displaced from plane of focus. Additional dumbbells are then “wound” around the cross’s peripheral BHs (**i**,**j**). The 3D assembly is then let go by the HOTs (**k**) and is able to maintain its own structure. 3D schematic for the final assembly is shown in (**l**).

**Figure 4:**
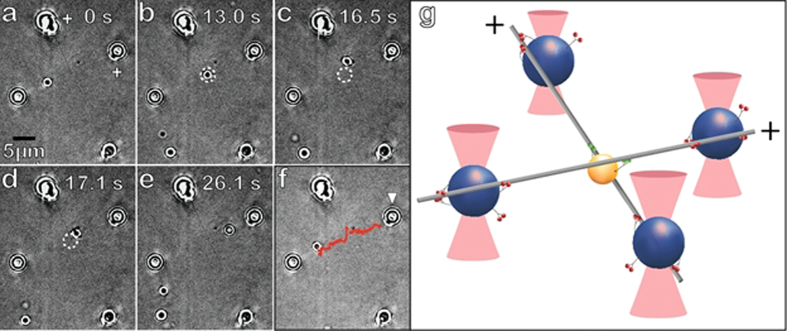
Technique validation for motility assays. (**a**) A MT crossing with 500 nm vertical filament separation is shown. An MC starts out progressing towards the intersection on the overlying MT. (**b**) MC reaches intersection and a tug of war ensues. The MC which is engaged on both MTs, starts to migrate up the transverse MT (**c**), but then after 0.6 seconds, disengages the transverse MT and snaps back (**d**) to once again solely translocate along the original MT (**e**). (**f**) Trace of the MC’s progression (red) overlaid on the original frame of the sequence shown in (**a**). This event is representative for assays with MC-MT binding fraction slightly below saturation. White arrow in (**f**) indicates the BH whose position is tracked (Supplement Fig. 6). (**g**) Graphical model (not to scale) depicting the key aspects of the set-up in (**a**–**f**) and tug of war scenario in frame (**c**).

In this work we manipulate MTs devoid of artificially induced chemical modifications, or MT-associated proteins, to retain maximum flexibility in modeling MT-based transport and biomechanics (chemical complexity can be introduced via a straightforward alteration of our assay). We orient individual MTs by tethering refractive microspheres (hereafter bead handles or BHs) along each filament (Fig. 1) which can then be manipulated in 3D via HOTs to construct complex 3D MT networks (Figs 2 and 3). Model cargos with enzymatically active motors (hereafter motorized cargos or MCs) and chemical factors regulating their activity may be incorporated to study transport on these networks (Figs 1 and 4). This strategy presents several challenges: (1) BHs need to have robust affinity for MTs so that adsorption onto MTs is efficient and stable; (2) BHs with many high affinity MT sites on their surfaces may serve to cluster MTs, so special care must be taken to ensure MTs can be manipulated individually. Below we describe how each challenge was addressed in detail.

*(1) BH-MT affinity*. Silica is our chosen BH material due to its high density (see below). We biotinylate the BHs’ surface (Biotin-PEG-Silane) and then attach biotin-tris-NTA via a neutravidin bridge (Fig. 1b). Finally, we specifically attach a full-length enzymatically dead kinesin-1 mutant (E237A in hKIF5A) with a C-terminus HIS6 tag to confer BH-MT affinity (Fig. 1a–c). BH surfaces can be functionalized with various amounts of E237A kinesins to tune the robustness of BH-MT attachment.

*(2) BH-MT clustering*. BH and MT concentrations can be tuned to prevent BHs and MTs from forming unintended mesh-works (Supplementary Video 1) during mixing. Optionally, BHs and MTs can be co-incubated briefly on a nutator to facilitate occasional BH-MT association (Figs 1d and 2a). Long term separation between these components within the flow cell is achieved by using high density materials for BHs (e.g. silica). Upon admission into the flow cell, BHs sink while MTs float, thus quickly and reliably separating these otherwise coalescing species. Note: coverslip surface must be blocked well to prevent BH and MT coverslip adhesion (see Methods).

Ideally, a flow chamber will exhibit sparse MT coverage with about 2–3 BHs per field of view. As discussed above, it is often easiest to start network construction by capturing a BH pre-attached to a MT (Fig. 2a) in one HOT. A standalone BH may then be captured by an independent HOT and held near the MT at a desired displacement from the already attached BH (Fig. 2b). Firm attachment is quickly observed for BHs functionalized with high amounts of enzymatically dead kinesin. We refer to BH-MT-BH assemblies as “dumbbells” due to their shape. Dumbbells are tested for structural integrity and are then moved by repositioning one or both of the BHs with a HOT (Fig. 2c).

It is often desirable to ascertain that two BHs are connected by a single MT rather than a MT bundle, however doing so via direct imaging often proves difficult. A third “reporter” bead may then be attached to the dumbbell a few microns away from the BHs (Supplementary Fig. 1) and its diffusion can be analyzed (Supplementary Fig. 2) to evaluate the stiffness of the dumbbell’s backbone, similar to previous approaches^22^. This technique is convenient enough in practice to be used *in situ* as a generic quality control step. Other optical trapping based techniques^23^ are also compatible with our setup.

Our assay also allows mechanical properties of filament networks to be varied. For example, BH-MT linkage stiffness can be tuned by varying the number of motor cross-bridges and/or the nature of the cross-bridges (e.g. using shorter motor constructs). Alternatively, mechanical and chemical properties of MTs could be adjusted by varying tubulin polymerization conditions or adding MT-associated proteins^24^. Notably, mechanical properties of individual MTs can be easily pre-screened (Supplementary Fig. 1,2), so that mechanically heterogeneous networks can be easily designed and built with our approach.

To increase network construction efficiency, building blocks (free BHs, single beads with an attached MT, or whole dumbbells) can be captured and/or fabricated anywhere within the flow cell and then stockpiled in a designated location for subsequent use. An added benefit of silica beads is their propensity to remain relatively stationary upon settling to the surface. This allows all building blocks to be stored in designated areas. A sample stage equipped with absolute position gauge with micron-scale readout precision facilitates repeatedly accessing the stockpile location.

To study transport across complex 3D MT networks, MCs can be added to the assay buffer (Fig. 1a,c,d) prior to admission into the flow cell (here: silica beads with WT hKIF5A motors). Beads of significantly different diameters are used to distinguish MCs from BHs. MT polarity for each dumbbell is readily determined *in situ* by observing the motility direction of an MC driven by a known motor type, e.g. kinesin-1 (Fig. 2d–g).

To demonstrate basic dynamic network construction, we depict assembly of a 3D MT-MT intersection - the fundamental unit of network complexity. Two dumbbells are stretched taut. One MT (horizontal MT, Fig. 3a and Supplementary Video 2) is raised high enough to move over the perpendicular MT without forming inadvertent BH-MT attachments (Fig. 3b), since BHs bind MTs nearly instantaneously on contact. After MT-MT cross formation, filament separation and intersection angle can be freely adjusted (Fig. 3c,d). Z-axis positioning is easily calibrated (Supplementary Fig. 3,4) and positioning precision of 50 nm is reliably achieved. This is comparable to or better than the relevant *in vivo* length scale^16^.

Our approach also allows us to cross-link MT filaments into stable 3D arrangements, i.e. self-supporting 3D networks. Figure 3e–l demonstrates this capability. A MT cross (Fig. 3g) is initially deformed into a dome shape by HOTs (Fig. 3i). Additional dumbbells are then used to cross-link the base of the dome structure (Fig. 3i, j), resulting in a dome that maintains its shape without HOT support (Fig. 3k,l). This geometry was chosen for demonstration purposes because it is visually simple, while displaying the key features: all MTs are substantially bent so the integrity of the structure is immediately apparent upon inspection and the shape has significant extent in all three dimensions (no single plane intersects all the beads in this network).

The construction capabilities we show above are fully compatible with most established *in vitro* motility assay types (we also envision construction of 3D filament structures as a scaffolding for cell culture experiments). For example, kinesin-1 motors show robust motility when moving along a MT suspended above the surface (Supplementary Fig. 5a). More complex experiments, e.g. studies of kinesin inhibitors are also easy to implement. Kinesin motility can be partially inhibited, for example by the addition of ATPγS (Supplementary Fig. 5b). The resultant magnitude of inhibition we observe is similar to what is expected from previous reports^25^,^26^. In fact, our approach is advantageous over techniques using MT attachment to glass slides even for the simplest motility assays because cargos (artificial or *ex vivo*) can be positioned far away from the glass slide, eliminating concerns regarding non-specific cargo-glass interactions, or variations in biophysical or chemical parameters near the surface.

A model *in vitro* transport experiment (Fig. 4 and Supplementary Video 3) demonstrates the additional advantages of our approach to enable more complex motility experiments. An MC is maneuvered (via an independent HOT) to engage one of the MTs making up a pre-built 3D MT-MT intersection. The MC attaches to a MT far from the crossing site and proceeds through the intersection with a clearly observable tug of war event (Fig. 4a–f). Characterizing the forces involved in such low motor number tug-of-war events is straightforward in our suspended MT system by examining BH displacement in their respective HOTs (Supplementary Fig. 6); this is essentially impossible with prior techniques. In our system, we routinely observe teams of kinesin motors (on a single cargo) engaging in a tug of war when interacting with transverse MT filaments at an intersection. These events are common *in vivo* and play a significant role in cargo transport at MT intersections^21^.

Such tug of war events affect cargo velocity and direction of motion, as well as the final outcome of the crossing events *in vitro*^9^ (e.g. whether the cargo proceeds along the original MT or switches to the intersecting filament). The same dynamics is likely important *in vivo*^16^. Intracellular transport cannot be fully understood without first quantitatively studying cargo routing in a baseline environment free of confounding cellular regulatory factors^7^. Our ability to characterize forces associated with tug of war events in our system is therefore essential to accurately model the general problem of cargo routing. A more detailed study is clearly desirable but is outside the scope of this work.

## Discussion

Our technique has two key features which set it apart from existing approaches: 1) the ability to control individual filament positioning in 3D with high fidelity and 2) the ability to incorporate multiple filaments to create complex 3D MT networks. Our approach is not only useful for motility assays and biomechanics experiments but can also be used to “wire up” nanodevices for molecular motor based transport e.g. 3. For example, some proposed biosensor designs^3^ rely on molecular motors to transport targeted analytes throughout distinct microchip compartments in a bead assay configuration. This concept has been validated^3^, but an inability to design definitive MT networks has hampered the technology’s progress. Our method finally enables the construction of arbitrary MT networks to route cargo in a customized manner. Our approach can also be easily integrated with other experimental protocols, which for example, may allow investigations of the effects of MT-associated proteins on motor-driven transport in the context of complex 3D MT networks.

Holographic technology is limited by the rate at which trap positions can be updated. This precludes using the HOT technology for experiments such as probing elastic moduli of networks over a wide frequency range^27^, or as force clamps^28^. This shortcoming has been overcome in our system by adding a conventional trap with fast repositioning capability to the optical train^18^, but at the expense of increased setup complexity.

Another limitation of our system is that construction of structures takes many minutes. Therefore, once constructed, a given network is likely to be reused for many experiments (e.g. motility assays). This allows experiments to be performed in a nearly identical environment which is usually advantageous for experimental design but also places a premium on the ability to visualize MTs without relying on fluorophores that photobleach quickly under intense illumination for a long duration. In addition, since MTs are likely to be arranged without alignment along one direction, we found conventional DIC imaging to be suboptimal for visualization. We have found that direct brightfield illumination imaging^29^ works best but our technique would benefit from extremely photostable labeling which could be demonstrated to not affect MT-based transport and elastic properties of the filaments. We stress that better visualization could help speed up the workflow and help scale the system toward more complexity, but this is not currently a critical limitation.

As we show above, our method is also well suited to create interconnected 3D MT geometries to construct shapes and engineering components. Mechanical properties of the filament building blocks can be altered by varying the number of motor cross-bridges, and/or the nature of the cross-bridges (e.g. using shorter motor constructs.) Mechanical properties of individual dumbbells can also be varied within the same assay^24^ and can then be easily pre-screened (Supplementary Fig. 1,2) prior to assembly, so that mechanically heterogeneous networks can be easily designed and built with our approach.

Our approach is highly scalable. A typical volume accessible in a single field of view is far larger than typical cellular volume. This potentially allows intracellular MT networks to be replicated *in vitro*, even allowing substantial homothetic magnification. Modern HOT control software (including our setup) allows for 100–200 traps to be simultaneously created and controlled, which is sufficient to construct many MT networks of biological and technological interest. Of course, it is already conceivable that this number of traps will present a limitation if highly complex networks become desirable. In addition, for high number of traps, network visualization will become increasingly difficult and it is likely that highly complex filament structures, such as micron-scale scaffolding or mechanical metamaterials, will require extensions of the technique to permit automated assembly.

In summary, due to its high precision and extreme scalability, our approach enables a wide range of experiments, from single molecule assays to large scale construction of rigid filament scaffolding or cellular scale transport networks. Similarly, many previously inaccessible biomimetic engineering designs become feasible with our system.

## Methods

### BH preparation

Monodisperse 2 μm silica beads 1% (w/v) (Microspheres-Nanospheres, Cold Spring, NY) were diluted by 20x in EtOH and were reacted with Biotin-PEG-Silane (MW 3400, Laysan Bio, Arab, AL). Biotin-PEG-Silane was added to a final concentration of 11.75 mg/ml per manufacturer recommendation. This amount of Biotin-PEG-Silane ensured we had at least 100X molar excess relative to reactive sites on the 2 μm silica beads (assuming 4.9 OH groups / nm^2^ for silica beads^30^). The beads were allowed to react for 1 hour at room temperature, then were washed 3 times in ultrapure water and were re-suspended in ultrapure water to maintain a 1% (w/v) bead concentration.

These biotinylated beads are incubated with an equal volume of 10 mg/ml of neutravidin (Thermo Fisher Scientific, Waltham, MA). The high molar ratio of neutravidin:surface biotin groups (~10:1) ensures that biotin groups are saturated with neutravidin. For daily use, an aliquot of the neutravidin-biotin-silica beads is incubated with 0.1 mg/ml of biotin-tris-NTA, a trivalent NTA derivative crosslinker (Biotechrabbit, Hennigsdorf, Germany) for 15 minutes. Assuming that each neutravidin has three available binding sites after being attached to the bead surface via a biotin ligand, the molar ratio of biotin-tris-NTA : neutravidin sites was at least 10:1. These beads, referred to hereafter as tris-NTA beads, are then activated with a 3X molar amount of NiCl_2_ or other metal salt such as CoCl_2_. After a 5 minute incubation, the Ni-NTA beads are diluted 7.5x in BH buffer (35 mM PIPES, 5 mM MgSO4 pH 7.2, supplanted with 1 mM GTP, 2.67 mM ATP, and optionally 40 μM Taxol.) Immotile E237A hKIF5A (full length HC construct, C-terminal HIS6 tag) is then added to a desired concentration (50–70 nM typically), and incubated for 30 minutes. Tris-NTA to HIS6 binding is reversible via the addition of various metal chelators or imidazole^31^, which is useful for control experiments.

In summary, our strategy is to saturate previously created surface functionalizing binding sites at every step, and then use as little E237A hKIF5A as functionally necessary (a generally applicable consideration, considering that E237A hKIF5A is effectively a custom made reagent). Our kinesin to glass surface attachment strategy is similar to the one described by the Surrey lab^32^.

### Motorized Cargo Preparation

Monodisperse 1 μm silica beads 1% (w/v) (Microspheres-Nanospheres, Cold Spring, NY) are diluted 20x in MC buffer (35 mM PIPES, 5 mM MgSO4 pH 7.2, supplanted with 5mM ATP, 5 mM DTT). The use of silica for MCs ensures that the model cargo will have a preferred downward orientation when attached to a MT. Bead materials with density matched to our buffer result in more diffusive MC motion. The desired concentration of wild-type hKIF5A (full length HC construct, C-terminal HIS6 tag) are then added to the motorized cargo buffer, and incubated for 30 minutes. Casein (40 mg/mL stock; 100X final dilution; MP Biomedicals) is added with the motors to block the surface and reduce clumping.

### Assay Buffer Preparation

Due to surface passivation, adding components sequentially will displace materials administered prior. Therefore, BHs and MTs are combined and incubated for 2 minutes. Thereafter MCs are added to the solution. The volume ratio of BH:MC:MT components is 85:10:5. The resulting mix is then quickly admitted into the flow cell.

### Coverslip Silanization

#1.5 Borosilicate coverslips (VWR, Radnor, PA) are subjected to a chemical etch in Piranha solution for 1 hr. Coverslips are then washed 3 times in ultrapure water and then are dried in an oven for 1 hour at 100 degrees. The coverslips are then incubated in a beaker containing 10% Hexamethyldisilazane (HMDS) (Gelest, Morrisville, PA) solution in toluene for at least 1 hour (overnight is better). These HMDS-treated coverslips are then washed 3 times in EtOH, once in ultrapure water and are then cured in the oven at 100 degrees for 1 hour.

### Flow Chamber Preparation

A flow chamber is constructed by attaching an HMDS-treated coverslip to a coverslide via parallel strips of double-sided tape (3 M, Saint Paul, MN). 1 flow chamber volume of 10% pluronic F-127 (Sigma-Aldrich, St. Louis, MO) in ultrapure water is admitted and allowed to incubate for 15 minutes. Then 2 flow chamber volumes of casein (40 mg/ml in PMEE) is administered and allowed to sit for 15 minutes.

Our approach is similar to the one described previously^33^ however we find that additional blocking with casein improves surface blocking performance. Many similar techniques have been developed e.g. 33,34. They are likely to provide similar or better performance. However, casein blocking alone, even using κ-casein isoform exclusively, does not provide acceptable blocking for our assays.

### Optics and imaging

Imaging was performed using a Nikon Eclipse-Ti microscope equipped with a high-magnification, high-NA objective (Nikon Plan Apo VC 100 oil, 1.40 NA). A high-resolution camera (iXON DU897; Andor Technology USA) and Nikon NIS elements AR software (Nikon Instruments USA, Melville, NY) were used to record experiments. Typical field of view size for this work was ~55μm × 55μm.

The holographic optical trap system was set up as previously described^18^. Briefly, a spatial light modulator (Boulder Nonlinear Systems, Lafayette, CO) was installed in an optical plane conjugate to the back focal plane of the objective. BioRyx software (Haemonetics, Braintree, MA) was used to define and control holographic trap arrangement.

### Microtubule Preparation

Porcine tubulin (Cytoskeleton, Denver, CO) was polymerized according to the manufacturer’s protocol to produce MTs with a tubulin concentration of 140 μM. All MTs used in this work were taxol-stabilized. Polymerized MTs are then diluted 100x in MT buffer (35 mM PIPES, 5 mM MgSO4 pH 7.2, supplanted with 1 mM GTP, and 40 μM Taxol.)

### Kinesin Purification

hKIF5A KHC dimers (wildtype sequence with HIS6 tag on C-terminus) were expressed in an E. coli system as previously described^35^. Briefly, full length KHC with N-terminus MBP solubility tag was expressed and purified. The MBP tag was then proteolytically cleaved as a final purification step.

## Additional Information

**How to cite this article**: Bergman, J. *et al*. Constructing 3D microtubule networks using holographic optical trapping. *Sci. Rep*. **5**, 18085; doi: 10.1038/srep18085 (2015).

## Supplementary Material

Supplementary Information

Supplementary Video S1

Supplementary Video S2

Supplementary Video S3
