# Supplementary material for: Constructing 3D microtubule networks using holographic optical trapping
Source: Sci Rep. 2015 Dec 10;5:18085. doi: 10.1038/srep18085 (PMC4674800; doi:10.1038/srep18085)
Supplement: Supplementary Information [file srep18085-s1.pdf]

Supplementary Information for

Constructing 3D microtubule networks using holographic optical trapping

J. Bergman, O. Osunbayo, M. Vershinin*

Department of Physics & Astronomy,
Department of Biology,
Center for Cell and Genome Science,
University of Utah,
Salt Lake City UT 84112

**e-mail address: vershinin@physics.utah.edu*

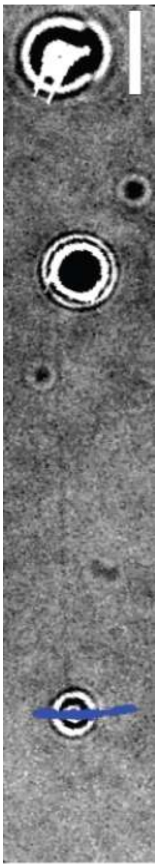

Supplementary Figure 1: MT stiffness assay. MTs were held in the imaging plane via two BHs spaced several microns apart and held in holographic optical traps at maximum displacement to assure no slack in the MT. The free end of the MT was then allowed to move under the influence of Brownian fluctuations. A BH acting as fiduciary marker was then placed on the MT to report MT displacements. Bead displacements were predominantly in the transverse direction (blue). Scale bar: 10 μm.

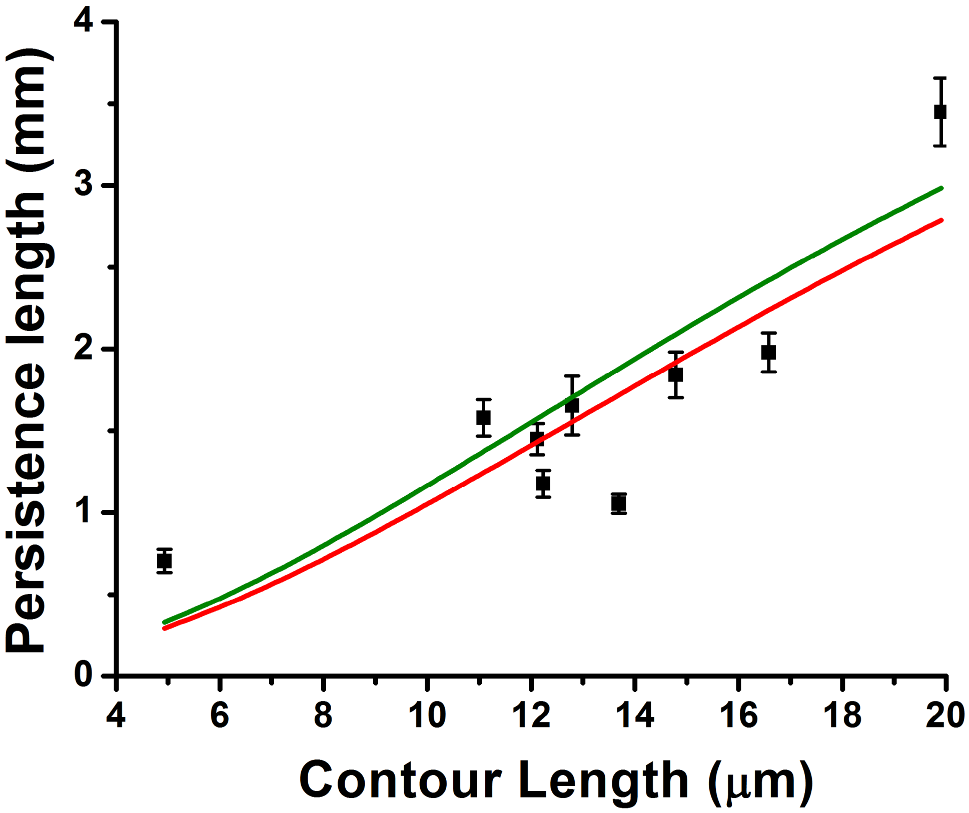

Supplementary Figure 2: MT stiffness analysis. The data acquired as shown in Supplement Figure 1 was analyzed similar to Pampaloni et al. (ref. 20, main text). Though MT lengths in our experiments were not sufficient to accurately estimate saturation level for persistence length of MTs, our data fits well to eqn. 4 in Pampaloni et al. if this parameter value is set at 6.3 mm (red curve). The cross-over length for our data (22.4 ± 1.3 μm) is close to the previously reported fit (green curve).

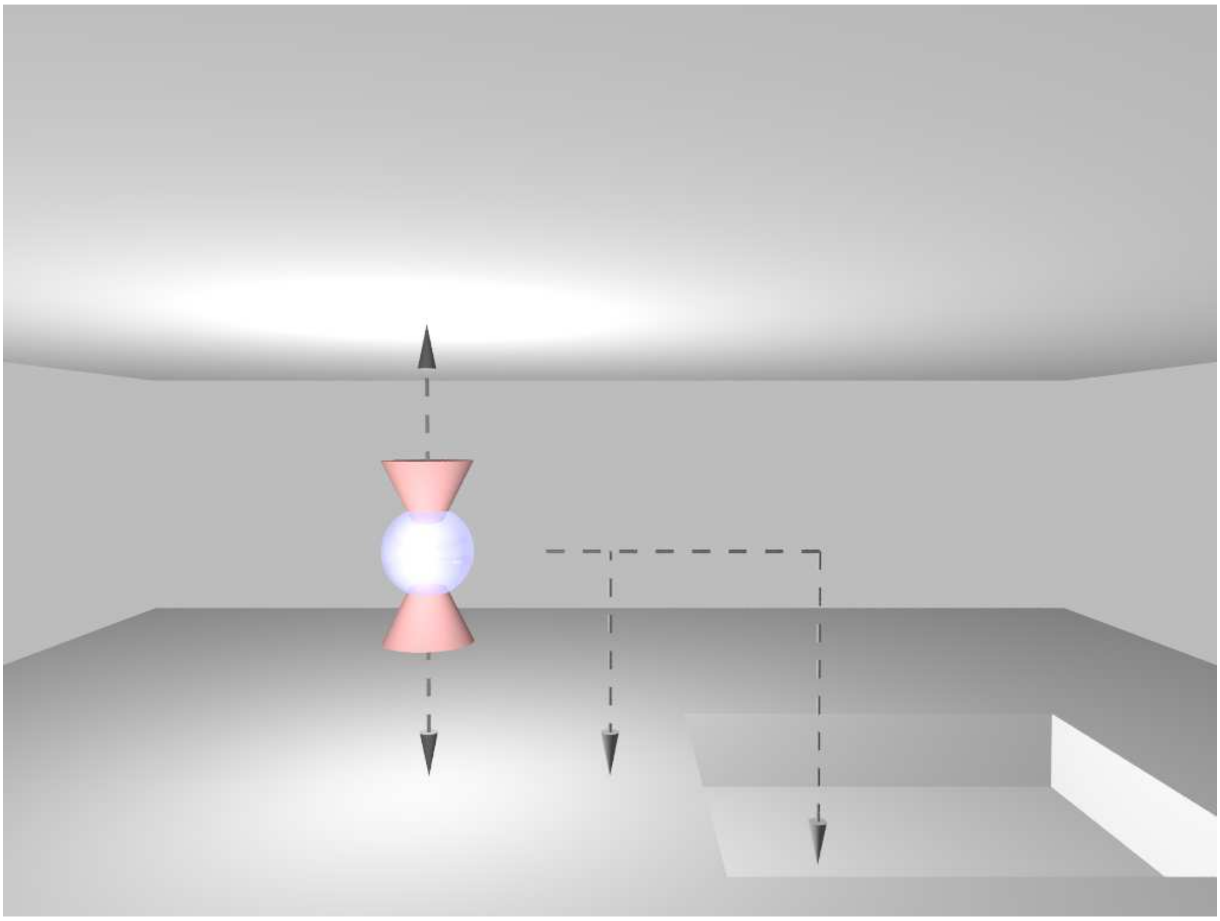

Supplementary Figure 3: Z-axis calibration. Unlike X and Y bead displacements (i.e. movement along axes orthogonal to the microscope axis), the Z-axis displacement cannot be estimated directly from video record. To calibrate Z-axis positioning, we have raised and lowered a NIST traceable silica bead (Microspheres-Nanospheres, Cold Spring, NY) in a holographic optical trap until the bead touched the bounding surface of the flow cell. In one method, the bead was limited by the upper and lower flow cell walls separated by a known distance (left dashed lines and arrows). 10µm polystyrene beads (CV=5%) (Polysciences, Warrington, PA) were typically used as spacers though other spacers (e.g. 5 µm thick double sided tape 68556, TESA, Charlotte, NC) produced nearly identical calibration values. We calibrated the HOT software Z-axis positioning units (i.e. voxel height) by dividing the bead probe's range of motion by the corresponding number of voxels. In another method, we have used focused ion beam to etch a flat bottom trench or a staircase (supplementary figure 4) into a coverslip. Etched trench depths were measured with an atomic force microscope and used as reference distance for voxel height calibration (right dashed lines and arrows). With either approach, when the focal plane of the objective coincided with the bottom plane of the flow cell our voxel height was 131 $\pm$ 2 nm (100X NA oil objective). The calibrations for other focal planes of the objective were within 10% of this value.

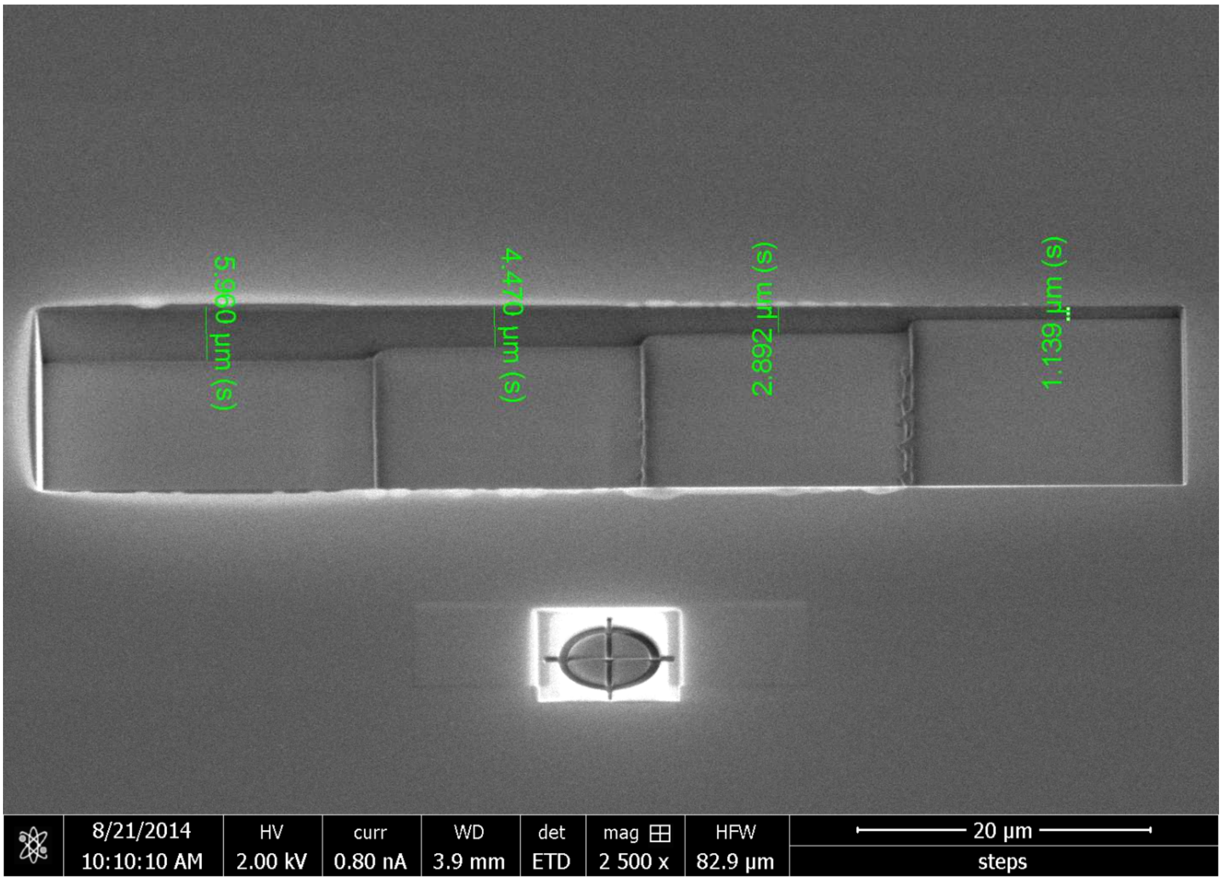

Supplementary Figure 4. Example of 3D coverslip steps used to calibrate voxel height. Focused ion beam etching was used to create step structures which could then be used for Z-axis calibration (Supplement Fig. 3). Steps of ~1µm height were typically used.

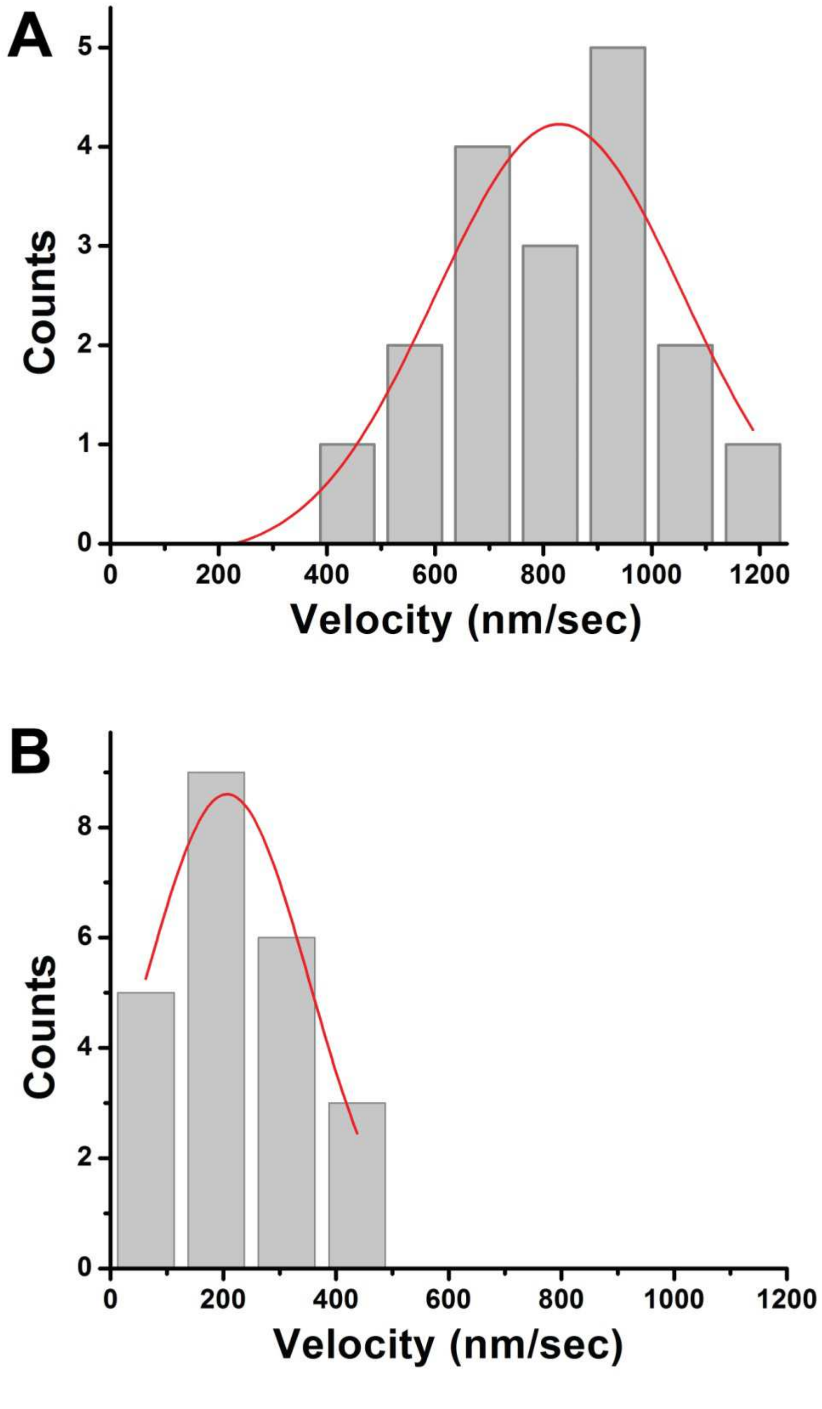

Supplementary Figure 5. Motility assays on suspended MTs. Healthy motor velocities (814±45 nm/sec; n=18; mean±SEM) are seen for kinesin-1 driven MCs (A). However, adding ATPγS inhibitor (ATP: ATPγS molar ratio 2.25:1) reduces the velocities significantly (233±117 nm/sec; n=23; mean±SEM); t-test $p<10^{-13}$.

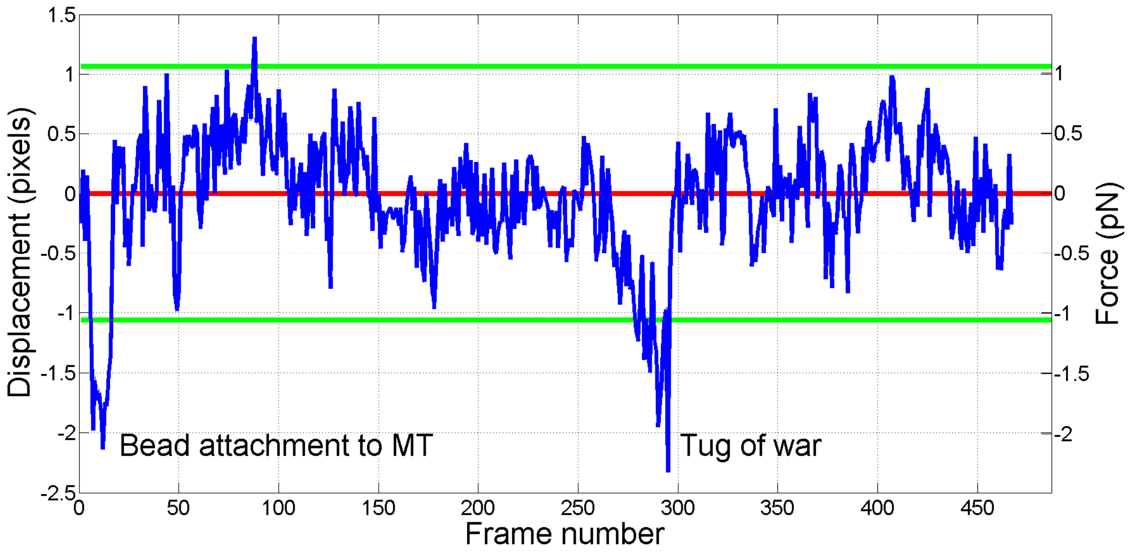

Supplementary Figure 6. BH tracking and force readout. Tracking of the position of the upper right BH in Fig. 4 and Supplementary Video 2 (bead is highlighted by arrow in Fig. 4f). The bead generally shows limited diffusion around its median bead position (red line) and instantaneous bead position is typically within 2 standard deviations of the median (green lines). However two sustained deviation outside of these limits are observed. First occurs when an MC is placed on the MT connected to the BH being tracked. The force is exerted on the BH because the MC is initially moved by the HOT resulting in a perturbation when the MC engages on the MT. The second event occurs during MC navigation at the intersection and provides direct readout of the tug of war forces involved in the process. Here the maximum pulling force of 1.4 pN is observed (107 nm/px imaging; 0.95 pN/100 nm optical trap stiffness).

Supplementary Video 1: MT asters on BHs can be induced via prolonged BH co-incubation on a nutator with an excess of MTs. Such asters are easy to discern during an experiment. The video is included for contrast with the controlled assembly we achieve when prolonged co-incubation is avoided.

Supplementary Video 2: Assembly and dynamic adjustment of MT cross arrangement shown in Fig. 3.

Supplementary Video 3: Kinesin-1 driven bead traverses a MT crossing as shown in Fig. 4.

Supplement Text 1: As discussed in the main text, it may be desirable to ascertain that dumbbells consist of two beads connected by a singular MT rather than a MT bundle. In practice, a typical dumbbell we construct is >10 μm in length. Most MTs we and others produce via polymerization under normal conditions are much shorter than this [1]. Therefore the probability of two extremely long MTs being aligned between two beads is very low. A greater concern is that it is difficult to see if shorter MTs are present near a long MT which is linking the dumbbell together. Shorter MTs will not cross-link the dumbbell but may interfere with 3D structural assembly or motility experiments. To test for these unwanted conditions, a third bead may be placed on the dumbbell as shown in Supplement Figure 1. One can then track the displacement of the marker bead (lowest bead in Supplement Figure 1) while holding the other two beads trapped. With this data, one may then conduct MT flexural rigidity analysis as shown in Supplement Figure 2 and evaluate the hypothesis that the persistence length of the dumbbell is on par with a single MT, or a bundle of MTs.

Note that most of the persistence lengths in Supplement Figure 2 are for contour lengths between 11 and 17 μm and the standard deviation for this data is ~0.33 mm (if one subtracts the fitted trend then the standard deviation becomes ~0.32 mm so that our estimate of overall noise is not notably skewed by the slope in the trend). If two MTs were connecting the dumbbell (and therefore if two MTs were diffusing together as one unit) then the second moment of MT cross-section would at least double (greater increase is likely depending on the relative geometric arrangement of the two MTs) leading to a commensurate increase in the observed persistence length. The data uncertainty is likely to add in quadrature leading to a standard deviation estimate of ~0.47 mm. Therefore, we propose discarding all dumbbells (for this range of contour lengths) with persistence length above 2*6.3*(1+(22.4/L)^2)-2*0.47 where L is contour length. In other words, we propose discarding all dumbbells whose persistence lengths are higher than or within two standard deviations of double the fitted trend. More data for the very longest dumbbells will be needed to establish that this criterion works in those cases. However in practice, all dumbbells we have tested to date have passed such a test. As expected, the probability of doubling up of MTs for dumbbells above 10 μm in length is a very unlikely possibility and this likelihood will only decrease as the dumbbell length increases (due to a decrease in number of very long MTs). Thus, such occurrences appear to be of negligible likelihood for the longest dumbbells (>20 μm) and may not be worth explicit testing.

Another consideration yields similar conclusions, though less directly. If we assume that doubling up of MTs in a dumbbell occurs randomly, then half of such dumbbells would be cross-linked by oppositely oriented MTs. In that case, kinesins on MCs would be free to engage on both MTs and such engagements would produce observable tug of war events [2]. Therefore, the healthy unidirectional MC motion routinely observed in our work further argues that dumbbells crosslinked by two or more MTs are negligibly rare.

Supplement Text 2: The development of this technique took several years and was interrupted several times due to technical difficulties. We discuss some of these difficulties below in hopes it will help other implementations of our approach. By far the biggest problem for us was our strict requirement that MTs must remain unmodified except where BHs bind to them. For example, we avoided using biotinylated MTs because we could not envision a way to ascertain that the MT-bound biotins would not interfere with the binding of any MT-associated protein under any conditions or that they would not affect any MT mechanical properties. This put a premium on finding a good agent to put on BHs to serve as “glue”. Our trials of a vast number of antibodies have all been fruitless. All antibodies we tested would “slide” along MTs under applied load. Incubating beads with extremely high amounts of antibodies would result in bead clumping. Similarly many MAPs may slide along MTs under applied load and cannot be relied upon to provide a robust fixed attachment point between a bead and a MT, consistent with recent reports [3].

In addition, we desired a “glue” which would allow for nearly instantaneous binding as soon as bead and MT were brought near each other because any significant delay could affect structure assembly rate and binding reliability especially for larger structures. Therefore, our requirements placed a premium on molecular solutions where the binding site was flexibly attached to the tag which we could use to functionalize our BHs. The recombinant expression of enzymatically dead kinesin-1 HC dimers fulfilled all our requirements, although efficient expression of full length kinesins without aggregation initially proved very challenging.

The problem of separating BHs and MTs to prevent aster formation was comparatively easier. We started with polystyrene bead BHs and used extremely high bead and MT dilutions so that it often took us minutes of searching across the flow cell to find any MTs and beads. At such low dilutions, simplest structural assemblies were possible so the proof of principle was obtained early. However, extremely low dilutions and extremely long searches were not practical and moreover polystyrene beads were hard to store for later use because they diffused too readily. We therefore switched to silica beads which readily sunk to the bottom. However, casein-blocked surfaces were not blocked well-enough and beads would bind to the surface after some time. We therefore developed better surface passivation techniques which solved our issues with the storage of prebuilt components for later assembly. Through routine use of such assays we discovered that silica beads sunk fast enough that they could be mixed with MTs for a short time, then admitted into glass slides and they would efficiently spatially segregate from the MTs if MTs were at a low enough concentration as described in the main text. This finally made all the pieces we needed readily available for fast and efficient workflow.

The final challenge for us was finding a way to reliably attach enzymatically dead motors to beads. This is essential because if the attachment is not robust then such dead motors may detach from BHs and reattach along MTs. This was observed early on and efforts were made to improve bead to motor attachments.

As the technique stands today, many MT crosses and similar simple 3D structure can be assembled and used in one day by one operator. The typical assembly time for such simple structures is a few minutes

for a trained experimentalist. The workflow is convenient and practical, especially since many experimental designs allow complex 3D structures to be subjected to many repeated experiments.

References Cited:

1. Lin, Y.-C., Koenderink, G. H., MacKintosh, F. C. & Weitz, D. A. Viscoelastic Properties of Microtubule Networks. *Macromolecules* **40,** 7714–7720 (2007).
2. Osunbayo, O. *et al.* Cargo Transport at Microtubule Crossings: Evidence for Prolonged Tug-of-War between Kinesin Motors. *Biophys. J.* **108,** 1480–1483 (2015).
3. Hinrichs, M. H. *et al.* Tau Protein Diffuses along the Microtubule Lattice. *J. Biol. Chem.* **287,** 38559–38568 (2012).
